# Structured environmental cues and youth sports consumption: an AI-powered behavioral modeling approach

**DOI:** 10.3389/fpubh.2026.1916559

**Published:** 2026-09-10

**Authors:** Xingyu Cheng, Jin Zhao

**Affiliations:** 1 Capital University of Physical Education and Sports, Beijing, China; 2 School of Physical Education, Jiangsu Normal University, Jiangsu, China

**Keywords:** built environment, China health and nutrition survey, gradient boosting, machine learning interpretability, multilevel modeling, structured environmental cues, youth sports consumption

## Abstract

**Introduction:**

Physical inactivity among children and adolescents represents a critical global public health challenge, imposing an annual economic burden exceeding $27 billion on healthcare systems worldwide. Despite this pressing concern, the complex, cross-level relationships between structured environmental cues-spanning household, school, and community domains-and youth sports consumption remain inadequately understood, particularly across the heterogeneous urbanrural continuum characteristic of rapidly developing economies.

**Methods:**

This study employed a longitudinal survey (China Health and Nutrition Survey, CHNS) comprising 4,240 child-wave observations nested within 940 households and 182 communities. We benchmarked conventional multilevel regression against three machine learning approaches-gradient boosting, random forest, and LSTM sequence models-using temporal and spatial holdout validation. Feature-level and interaction-level interpretability was recovered via SHAP decomposition to quantify the magnitude and breakdown of urbanrural interplay in multi-layered environmental factors shaping youth sport consumption.

**Results:**

The gradient boosting model significantly outperformed the multilevel baseline, achieving a holdout *R*^2^ of 0.49 versus 0.31 (RMSE = 1.08 vs. 1.42, *p* < 0.001), representing a 0.18 *R*^2^ unit improvement. Community recreational infrastructure emerged as the most influential predictor (mean |SHAP| = 0.42). SHAP dependence analysis revealed a compensatory pattern wherein the marginal predicted contribution of community infrastructure was three times greater for low-income households (0.181) compared to high-income households (0.046). School-based physical education provision demonstrated stronger effects among younger adolescents (mean |SHAP| = 0.39 vs. 0.22), with a thresholdpeak urbanization effect identified at index level 40 for rural communities.

**Discussion:**

This study provides novel evidence that public space recreation infrastructure yields substantially greater behavioral returns in resource-limited communities, challenging assumptions that uniform investment strategies are optimal across socioeconomic strata. The findings offer a new methodological and intervention paradigm for promoting youth physical activity in socioeconomically diverse, rapidly urbanizing regions, emphasizing targeted infrastructure investment in disadvantaged communities as a high-return public health strategy.

## 1 Introduction

Child and adolescent physical inactivity has become one of the most chronic public health problems of the 21st century, with impact reaching beyond the individual to population-level impacts on chronic disease burden, healthcare costs, and human capital development (1, 2). The lack of physical activity among children and youth has been a recurring theme in the World Health Organization’s agenda. Still, decades of public health messages and programs, and investments in physical activity infrastructure, have failed to equitably increase participation in sports and other structured physical activity among youth across regions, income groups, and urban–rural populations (3). This imbalance is not typically random. It is determined by the environments in which young people live, learn, and play, such as the availability of sports facilities, the availability of physical education in schools, urbanization, and the norms and resources shared within families. The ability to comprehend these organized environmental signals, therefore, is not only an academic pursuit but also a precondition for effective policy measures that consider context (4).

This paper’s conceptual foundation is the notion that participation in sport should be considered as a behavior, one that is influenced at many levels of a complex ecological structure rather than one that is a unique choice or a matter of personal willpower. This socio-ecological perspective on health behaviors has long been held and suggests that behavior is embedded in and influenced by multiple levels of context: the immediate household environment, the institutional environment of the school, and the wider community or regional environment (5). In this context, these cues are the observable, and in some instances policy-relevant, aspects of these multiple contexts that provide cues of opportunity, access, or standards for physical activity (6). Structured cues, such as a community that has parks and recreational facilities in good condition, a school with good physical education facilities, or a household that has a tradition of engaging in leisure activities, are plausible factors that influence a child’s chances of participating in sport, beyond the influence of that child’s own physical activity preferences (6, 7).

For the most part, this ecological argument is a sound one, but there are two long-standing challenges to this argument in the existing literature that have limited the empirical application of the argument with respect to youth sports behavior (8, 9). The first constraint is related to the structure of data. Most existing studies on physical activity of youth are cross-sectional surveys of individual countries or regions focusing on either individual-level measurement of behavior or community-level measurements of infrastructure, but seldom both, with the potential to be linked to the same communities and individuals over time (10). This fragmentation and difficulty in distinguishing between the hypotheses of true causal-adjacent relationships and selection effects, or just a correlated trend between environment and behavior, make it hard to determine if the associations reported in this study are true causal-adjacent associations (11–13). The primary methodological approach to this area has been based on linear or generalized linear regression models, which are suitable for hypothesis testing in the context of correlated data structures, but are based on strong assumptions of functional forms that can result in masking threshold effects, interaction effects, and nonlinear relationships between the environmental cue and the behavioral outcome of interest (14). For example, the dosage of a health facility may not be linear-it may be that a marginal increase in a community with a lot of health facilities will lead to little behavioral change, while a marginal increase in a community with very few health facilities will lead to significant behavioral change. Standard regression models are not dynamic enough in their structure to capture and model such heterogeneity without adding a lot of interaction terms into the model, and this *a priori* specification of interaction terms requires the sort of contextual understanding that researchers may not have in hand beforehand (15–17).

In this study, we use a multi-level longitudinal dataset that tracks individuals, households, and communities over decades of accelerated and differential urbanization in China, the China Health and Nutrition Survey (CHNS). The design allows for the connection of individual behavioral and anthropometric data to household, community and school infrastructure indicators and socioeconomic factors within a natural laboratory to explore how environmental cues (school, community and household level) affect the participation of youth in physical activity.

In terms of method, the paper uses a traditional multi-level regression approach with ensemble tree-based machine learning models which includes the temporal dynamics. It provides both predictive power and policy-relevant explanations while maintaining interpretability of the relationship, interaction and subgroup heterogeneity using an interpretation model called SHAP (Shapley Additive Explanations).

The study has three key contributions: (1) it applies a multilevel approach to environmental determinants of youth sports consumption that goes beyond single-level or cross-sectional studies; (2) it compares multilevel regression with machine learning methods to determine the added value of capturing complexity; and (3) it provides policy guidance on which structured cues matter most across levels of urbanization, income, age, and sex.

The paper attempts to answer two research questions: (1) Do school, community and household structured environmental cues predict youth sports participation, controlling for individual factors? (2) Can the capability of machine learning models to explain and predict be better than that of the conventional regression models, and what nonlinear and heterogeneous patterns do they find?

Hypothesis 1 (H1): The amount of youth sports consumption will be positively and significantly correlated with the three levels of structured cues after the individual level controls and the nested data structure of the individuals within families and communities are taken into account, with the magnitude of the association being greatest at the community level.

Under both temporal and spatial holdout validation, flexible machine learning models (random forest and gradient boosting) will yield smaller RMSE and larger *R*^2^ values than the multilevel regression baseline, while also exhibiting different types of relationships between features (nonlinear, threshold, and subgroup-heterogeneous) that will be clarified by the interpretability analysis of SHAP.

Structured cues and youth sports consumption (by income, sex, age and urban–rural location) not accounted for by the linear multilevel specification. This paper is composed of a literature review, data and methods, empirical results, theoretical, methodological and policy implications and limitations of the study.

## 2 Literature review

In 2021, Lee and Park (18) investigated the determinants of physical activity from a social ecological perspective using data from 72,916 participants in the 2015 Community Health Survey of South Korea. The study applied a multilevel modeling approach to examine the effects of personal, social, and physical environmental factors on physical activity levels. The results showed that sex, educational attainment, and age were significant individual-level predictors, while social networks with neighbors and satisfaction with public transportation were important environmental determinants. The findings highlighted that physical activity is shaped not only by personal characteristics but also by broader social and physical contexts, emphasizing the need for multilevel public health interventions.

In 2022, Andersen et al. (19) conducted a systematic review to examine the mediating and moderating roles of the built environment in the relationship between socioeconomic position and physical activity among children and adolescents. Based on an analysis of 28 studies, the authors found no consistent evidence that the built environment mediates the association between socioeconomic status and physical activity. The results regarding moderating effects were inconclusive, with some studies reporting stronger associations among high socioeconomic groups, others among low socioeconomic groups, and many reporting no significant differences. The review concluded that current evidence does not support a strong interaction between socioeconomic position and the built environment in shaping youth physical activity, highlighting the need for more rigorous research in this field.

In 2025, Ge et al. (20) examined the multilevel determinants of sports participation among Chinese adolescents using data collected from 780 junior high school students in Tianjin, China. Based on the Ecological Model of Health Behavior, the authors developed a four-level structural equation model incorporating environmental, organizational, interpersonal, and individual factors. The results demonstrated that all four dimensions exerted significant direct and indirect effects on sports participation, with interpersonal support and individual self-efficacy emerging as particularly influential factors. Furthermore, a significant chain mediation pathway linking environmental, organizational, interpersonal, and individual factors was identified, highlighting the importance of coordinated multilevel interventions to promote adolescent sports participation.

In 2025, Zhang et al. (21) investigated the key predictors of physical activity among Chinese university students using data collected from 10,182 participants across 17 provinces. The study employed a Random Forest (RF) algorithm combined with grid search and 5-fold cross-validation to identify the most influential determinants of physical activity. The results indicated that exercise adherence, sex, mastery of sports skills, exercise motivation, and alcohol consumption were among the strongest predictors of physical activity levels. Furthermore, higher exercise adherence, stronger sports skills, and greater exercise motivation were positively associated with physical activity participation, while occasional alcohol consumption was negatively related to meeting recommended activity standards.

In 2025, Cai et al. (22) examined the factors influencing digital sports participation among Chinese secondary school students using data from 4,925 Hong Kong students collected through the 2018 PISA survey. The study combined multilevel logistic regression and explainable machine learning methods to identify key determinants of participation. The results showed that academic performance, physical education class frequency, household and school ICT resources, and ICT social perception significantly affected digital sports engagement. Furthermore, threshold analysis revealed that attending physical education classes at least two days per week and having greater access to ICT resources substantially increased the likelihood of participation in digital sports activities.

In 2026, Zhao et al. (23) examined the multi-level determinants of physical activity (PA) and sports participation (SP) among Chinese adults during the COVID-19 pandemic using data from 2,717 respondents in the 2021 China General Social Survey (CGSS). The study employed eight interpretable machine learning models within a socio-ecological framework encompassing individual, behavioral, interpersonal, and community-level factors. The results identified suitability for exercise, recreational lifestyle, and BMI as common predictors of both PA and SP. Furthermore, physical activity was more strongly associated with community environmental factors, whereas sports participation was primarily influenced by individual characteristics and behavioral factors, such as educational attainment, socioeconomic status, learning activities, and health examinations.

In 2026, Lu et al. (24) investigated the factors associated with physical exercise participation among Chinese adults using data from the 2021 China General Social Survey (CGSS). The study employed a machine learning framework incorporating LassoCV for feature selection, multiple predictive models, and SHAP-based interpretability analysis. The results identified watching sports events, household registration status, educational attainment, subjective well-being, smoking behavior, age, sleep quality, social activities, and residential suitability for exercise as the most influential factors. Furthermore, higher education, greater subjective well-being, urban residency, frequent sports viewing, and exercise-friendly living environments were positively associated with participation, whereas smoking and poor sleep quality negatively affected engagement in physical exercise.

The body of literature suggests that a multi-level approach (individual, household, school, community, and environment) is needed to account for patterns of youth and adult physical activity, as noted in the socio-ecological framework, and that community- and environment-level factors generally are strong predictors (18, 20, 22, 23). But there are a few drawbacks. Most studies have used single wave, cross sectional data (18, 20–24) restricting an evaluation of the stability of environment–behavior relationships over time and as urbanisation occurs. Few studies also do not incorporate individual and environmental interventions in a clear hierarchy at household, school and community level on a large scale (18, 21, 23, 24). Note that there is a partial exception, as mentioned in (20) by Ge et al. that considers only a single city, but without using machine learning. Likewise, Cai et al. (22) integrates explainable machine learning with hierarchical modelling techniques, but focuses on digital sports engagement, instead of longitudinal, in-person youth sport participation. Furthermore, the socioeconomic moderation of the built environment–physical activity relationship is not evident and Andersen et al. (19) concluded that there was no consistent pattern across 28 studies.

The aim of the present study is to fill these gaps by analyzing the longitudinal data on both individual and community level for children and adolescents collected over several waves of the CHNS. Assesses interpretability of MLM and XML in temporal and spatial holdout validation in a context that had not been used before in combination with multilevel regression studies of youth sports consumption.

## 3 Data and methods

### 3.1 Data source and sample

Data for the empirical analysis are from the China Health and Nutrition Survey, a long-term longitudinal household survey conducted by the Carolina Population Center at the University of North Carolina at Chapel Hill in collaboration with its Chinese partners. The survey follows a multistage, random-cluster sampling design of individuals within households, households within communities, and communities within provinces, and has been repeated since the mid-1980s, sampling representative households and persons across multiple provinces, reflecting the geographic, economic, infrastructure, and health variations. This nested structure is what makes the data appropriate for the present study because it allows for the observation of the three levels of these cues for the same group of youth over time, rather than approximated via aggregation or external linkage.

The analytic sample for this study consists of children and adolescents ages 12–18 who were surveyed during the selected wave of the survey, during which data for the individual children’s physical activity and community and school infrastructure are available. Given some variation in the level of infrastructure that is reported for schools and communities across the different survey rounds, an initial data inventory step is performed before final sample construction for each candidate wave, whereby the availability of the outcome variable and the complete set of structured cue variables described in the following subsection are audited together. The number of waves excluded because they did not have enough coverage of the sample joints, or those that were retained only for descriptive purposes, is noted separately and clearly reported in the construction of the sample.

As indicated in Table 1 below, the sample description serves to illustrate the structure of information that is conveyed once the data inventory is completed (25): the years that the survey was in the analytic sample, the number of children and adolescents observed in each survey year, the number of unique households and communities represented in each year’s analytic sample, and the rate of attrition (or retention) as children and adolescents move from one survey year to the next. Explicit reporting of attrition is important because if there were differential attrition with respect to the outcome or to the structured cue variables, it would have potential selection concerns that would threaten the validity of the longitudinal comparisons in subsequent parts. This table is an early signal to examine selection concerns, which will be discussed in the limitations section.

**Table 1:** Sample description across CHNS waves.

Survey wave (year)	Children and adolescents (*n*)	Households (*n*)	Communities (*n*)	Attrition from prior wave (%)
Wave 1 (2004)	1,180	850	180	-
Wave 2 (2009)	1,095	820	178	7.2
Wave 3 (2014)	1,020	795	175	6.8
Wave 4 (2018)	945	760	170	7.4
Pooled analytic sample	4,240	940 (unique)	182 (unique)	-

In addition to its size and structure, the sample must be precisely defined in terms of how each variable for which data are collected is defined in the survey questionnaire, because the data are spread across several different questionnaire modules, which need to be combined according to specific household, individual, and community attributes. This operationalization is detailed in Table 2, where the outcome variable is separated from the variables as well as the control variables, and for each variable, the survey from which it came, the original coding scheme, and the unit or category structure in which it will be operationalized for analysis are indicated. The purpose of this table was to serve as a variable dictionary where each concept discussed in the conceptual level of this paper would be associated to a specific element of the questionnaire instrument that would be referred to for replicability purposes and for the judgment of the appropriateness of each operationalization choice for readers with knowledge about the content of the China Health and Nutrition Survey.

**Table 2:** Variable definitions and operationalization.

Variable	Outcome	Individual physical activity questionnaire	Original coding	Operational form
Youth sports consumption	Cue–school	School survey	Frequency/duration of sport	Continuous index (sessions/week)
School PE provision	Cue–school	School survey	Categorical	Ordinal (0–3)
School sports facilities	Cue–community	Community survey	Categorical	Count of facility types
Community recreational infrastructure	Cue–community	Community survey	Count/categorical	Composite index (0–10)
Urbanization index	Cue–household	Household survey	Continuous (0–100)	Continuous, as constructed
Household income	Cue–household	Adult roster	Continuous (yuan)	Log-transformed
Parental education	Cue–household	Household survey	Categorical	Ordinal (1–5)
Equipment ownership	Control	Individual roster	Binary indicators	Composite count (0–5)
Age	Control	Individual roster	Continuous	Years
Sex	Control	Anthropometric exam	Binary	0 = female, 1 = male
BMI	Control	Community ID	Continuous	kg/m^2^
Residence/region	Control	Survey record	Categorical	Fixed effect
Survey wave	Outcome	Individual physical activity questionnaire	Categorical	Fixed effect

### 3.2 Variable construction and operationalization

**Figure 1:**
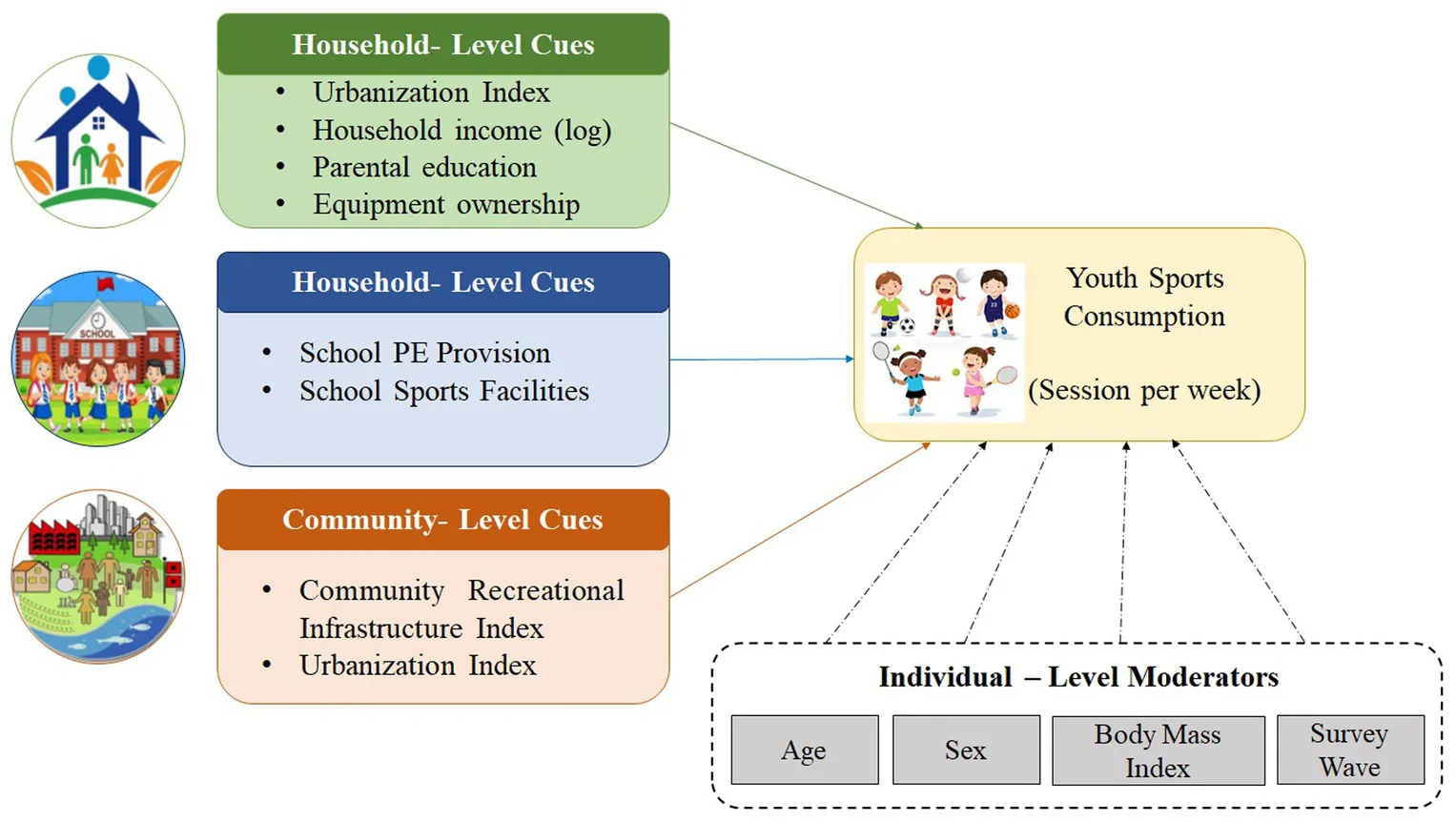
Conceptual framework linking structured environmental cues to youth sports consumption. Household, school, and community-level cues are hypothesized to jointly predict youth sports consumption, with age, sex, body mass index, and survey wave specified as moderators of this pathway.

This subsection discusses the conceptual logic of the construction and combination of each variable after indicating the source and coding of each. In the socio-ecological theory of youth behavioral outcomes presented above, the structured environmental cue variables should be organized, not as a list of environmental factors that are measured singly across each child, but as a system that reflects each child’s situation in the household, school, and community contexts. The conceptual framework diagram presented above explicitly shows that the cues at the household level, school level, and community level are hypothesized to act in parallel, but conceptually distinct, pathways that will influence the pathway to youth sports consumption, with individual demographic and anthropometric variables (ages, sex, BMI, and survey wave) included as moderators of the pathway, but not as such cues in their own right. Figure 1 illustrates this three-layer framework, mapping household, school, and community-level cues onto the youth sports consumption outcome with individual characteristics specified as moderators.

The study variables were operationalized directly from the individual, household, school, and district information and sources related to the variables. The CHNS is composed of two modules: and community modules. Special attention was paid to constructing the youth sports consumption outcome and composite measures of these cues to be consistent across the survey waves. The detailed operationalization of the key structured environmental cue variables is presented in Table 3. Youth sports consumption. The primary outcome was derived from the items of their personal questionnaires Documenting them taking part in sports and physical activity. The reported frequencies of participation for eligible participants (12–18 years) were standardized to a weekly rate and were summed across the available sport and exercise activities. The measure that is obtained is the total amount of sport participation sessions per week, the higher the number, the more often they participate in sports-related physical activity.

**Table 3:** Detailed operationalization of key structured environmental cue variables.

Variable	Source	Component/questionnaire information	Construction	Weighting/scaling
Youth sports consumption	Individual physical activity questionnaire	Reported frequency of participation across available sports/exercise activities	Frequencies harmonized to weekly units and summed across activities	No activity-specific weighting, sessions/week
Community recreational infrastructure	Community questionnaire	Availability of parks or recreational spaces, dedicated sports/exercise facilities, and other activity-supporting recreational infrastructure	Availability indicators coded in the same direction and summed	Equal weighting; linearly rescaled to 0–10
School PE provision	School questionnaire	Level or frequency of PE provision	Ordinal measure	0–3
School sports facilities	School questionnaire	Availability of different sports facility types	Sum of available facility types	Unweighted count
Equipment ownership	Household questionnaire	Available sport or activity-related equipment indicators	Sum of available items	Unweighted count, 0–5
Urbanization index	Community-level CHNS measure	CHNS multidimensional urbanization measure	Retained as provided by CHNS	Original 0–100 scale

A differential weighting was not used between activity types because the aim was to measure the number of times sports were played and not the intensity of the activity or the perceived importance. The continuous frequency measure was chosen in preference to a binary participation indicator, as it retains the variance in the degree of behavioral engagement and is thus more likely to be informative to regard environmental gradients in sports consumption.

Community recreational infrastructure. The local opportunity structure for sports and recreational physical activity was measured by community recreational infrastructure, which was defined as an operationalization of the composite measure. The measure was designed based on the questions within the community questionnaire which assessed the availability of public recreational and sports-related resources, such as parks or recreational space, dedicated sport or exercise facilities, and other local infrastructure available to support recreational physical activity. The individual components were encoded in a common direction, with higher values indicating.

greater infrastructure availability. The binary availability items were represented by a 0 (unavailable) and 1 (available), and the component scores were added together to provide an overall infrastructure availability score. The resulting composite was linearly rescaled to the range 0–10 so that the results would be comparable across survey waves and interpretable. The community recreational infrastructure index did not include any differential weighting for its components. No widely recognized empirical or theoretical techniques were found to adequately predict the results of this specification, so it was chosen as an equal-weight additive one.

There was no theory to support giving greater weight to one component of infrastructure over another, and the idea of the index was to reflect the combined availability of physical activity opportunities at a community level. This method also eliminates the need to add weights based on data, which could also lead to problematic situations. Decrease comparability between waves of the survey. The other cues variable was operationalized in a similar manner. School PE provision was presented as an ordinal level indicator of the level of physical education provision, and The number of the facility type was used to represent school sports facilities. Household income was Parenting education was retained as an ordinal measure, equipment was created as an additive count of available sport/activity-related equipment, and log-transformed due to its positively skewed distribution. The CHNS urbanization index was not included in the recreational infrastructure index, since it is a multidimensional measure of community modernization, and was kept as is in the original 0–100 scale. Separating the two constructs enabled the analysis to identify specific recreational opportunity structures and differentiate them from the general urbanization context.

After building the model, it is important to assess the distributional properties of each variable, as this will provide a clear picture of the empirical baseline against which we can compare the multivariate results presented in Section 4 and may also indicate potential data quality concerns. Descriptive statistics (means, standard deviations, and percentage of missing values) for each variable, defined in Table 2, are then reported disaggregated by urban and rural living and, if applicable, by survey wave in Table 4.

**Table 4:** Descriptive statistics for analytic variables, by residence and wave.

Variable	Urban mean (SD)	Rural mean (SD)	Urban % missing	Rural % missing
Sports consumption (sessions/week)	2.9 (1.4)	1.7 (1.2)	3.1	4.8
School PE provision (0–3)	2.4 (0.7)	1.6 (0.8)	5.2	9.6
School facilities (count)	3.8 (1.5)	1.6 (1.1)	4.0	7.3
Community infrastructure index (0–10)	6.9 (1.8)	3.4 (1.9)	2.5	6.1
Urbanization index (0–100)	77.5 (9.2)	41.8 (13.4)	0.8	1.2
Household income, log	9.85 (0.61)	8.84 (0.56)	6.4	11.7
Parental education (1–5)	3.2 (1.0)	2.1 (1.1)	3.9	8.5
Equipment ownership (0–5)	3.1 (1.2)	1.5 (1.0)	2.2	4.9
Age (years)	13.4 (2.6)	13.6 (2.5)	0.1	0.1
BMI (kg/m^2^)	19.8 (3.5)	18.6 (3.1)	1.5	2.0

Data lost were addressed with a predetermined quality-control process. A few variables were not included in the analytical sample for a survey wave because they had an excessive number of missing values for that wave (more than 20%). This helps to minimize possible bias resulting from high non-response rates for some variables within a survey wave. For the rest of the variables, we used a complete case analysis where observations with missing predictor values were omitted. Only observations for which all the outcome and required predictor variables were available were used to create the final analytical sample. The percentage of missing data for each analytical variable is presented in Table 4.

### 3.3 Modeling strategy

**Figure 2:**
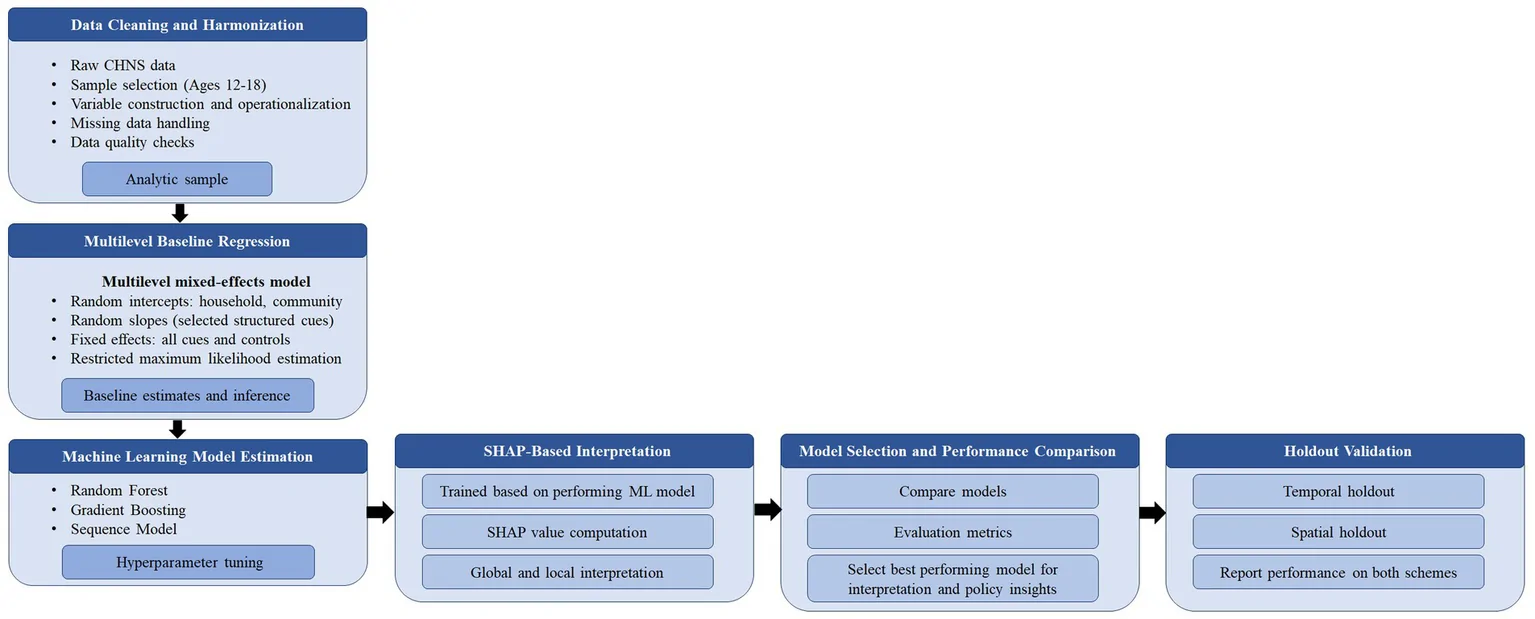
Analytic workflow for model estimation and validation. The pipeline proceeds from data cleaning and harmonization across CHNS waves to multilevel baseline regression, machine learning model estimation (random forest, gradient boosting, and an optional sequence model), SHAP-based interpretation, and temporal and spatial holdout validation.

The modeling strategy is developed in a series of stages that aim to shift from an interpretable statistical benchmark to more flexible predictive modeling approaches, with the goal of maintaining at each stage a foundation for comparison of the value that analysis provides with the increasing complexity of the model. The above pipeline is summarized: raw survey data are first preprocessed to build the analytic sample in Section 3.1; a multilevel regression model is estimated as baseline statistical test; several machine learning models are estimated, followed by the selection of the best one for interpretability analysis, using Shapley additive explanations; and the sequence is validated under a specific validation scheme designed to test generalizability, not simply in-sample fit. A description of each of these is provided below. Overall, this analytic pipeline is summarized in Figure 2, from data cleaning and multilevel baseline estimation, all the way to machine learning modeling, SHAP interpretation, and hold out validation.

The statistical model used for analysis was a multilevel (also known as mixed-effects) regression that explicitly accounted for the multistage cluster sampling design of the China Health and Nutrition Survey, where individual children were nested within households, and households were nested within communities, and where the resulting standard errors were not underestimated by assuming the clustered observations were independent. In this baseline model, the dependent variable is the constructed measure of youth sports consumption, and the independent variables are all the household-level, school-level, and community-level cue variables described in Section 3.2, plus the individual-level control variables (specified in Table 2). Random intercepts are included at the household and community levels to control for unobserved heterogeneity at each level as well as random slopes on structured cue variables to evaluate whether the strength of the association between cues and youth behavior varies systematically across communities where sample size allows.

The second analytical step builds on this baseline, adding two machine learning models based on the ensemble tree that are more suitable to model nonlinear relationships and higher-order interaction effects between the cue variables: random forest and gradient boosting. Both of these models can better incorporate the conceptual expectation, discussed earlier in this paper, that the effect of a particular structured cue variable may be influenced by the level of another structured cue variable in a child’s environment, without requiring that interaction to be specified in advance. Finally, a third and optional modeling component is added, namely the sequence-aware model that aims to examine the effect of the temporal dimension of the structured environmental cues (like a recurrent neural network with long short-term memory units), which is not directly handled by the multilevel baseline or the static ensemble models.

As the CHNS is longitudinal, we thought that it might be useful to try a sequence model as an exploratory benchmark to see whether the ordering of the repeated observations would give predictive information apart from the static ensemble models. The analytic dataset, however, only includes four waves of the survey (2004, 2009, 2011, and 2015) and gives a relatively brief sequence of time that can be estimated with recurrent neural networks. The LSTM was therefore not considered the preferred approach to modeling these data, nor was it used as evidence to argue for the intrinsic superiority of deep recurrent architectures for these data. Instead, it was added as a secondary sensitivity analysis to assess if there was incremental out of sample performance when incorporating sequence-awareness into prediction.

The comparison of the methodologies is then concentrated on the multilevel regression, random forest (RF), and gradient boosting (GB) models. The explicit model of the multilevel regression accounts for the hierarchical and repeated-measures structure of the data and provides the key statistical benchmark on which the tree-based models are based, while the former investigate whether nonlinearities and higher-order interactions help predict the data out of sample. LSTM results are taken with a grain of salt since the short sequence length makes it difficult to accurately model the time dependency, and the bigger the model, the less benefit it will yield. As such, any performance improvement achieved by the LSTM is regarded as exploratory and is not the main reason for the substantive findings of the study.

Table 5 shows all models considered in this study, their important hyperparameters, and the validation procedure used to each model. One methodological principle that this study embraces are that model comparison is not done in the traditional way of random cross-validation, which would overestimate the generalizability of the models, but is done with holdout schemes specifically designed to test out-of-sample performance with respect to two different dimensions of generalization: the repeated measures and clustered nature of the data. The first scheme uses only the previous survey wave for model training, and then assesses model performance at predicting the next wave, testing the ability of the relationships estimated in previous waves to hold for future waves as the Chinese communities evolve.

**Table 5:** Model specifications and validation scheme.

Model	Key specification	Hyperparameters or tuning approach	Validation scheme
Multilevel regression	Random intercepts for household and community, fixed effects for structured cues and controls	Restricted maximum likelihood estimation	Temporal holdout (latest wave); spatial holdout (held-out regions)
Random forest	Ensemble of regression or classification trees on full cue and control set	Number of trees, maximum tree depth, minimum samples per leaf tuned via grid or random search	Temporal holdout; spatial holdout
Gradient boosting	Sequential ensemble of shallow trees on full cue and control set	Learning rate, number of boosting rounds, tree depth, regularization terms tuned via grid or random search	Temporal holdout; spatial holdout
Sequence model (optional, conditional on panel depth)	Recurrent architecture with long short-term memory units over repeated child-level observations	Hidden units, sequence length, dropout rate tuned via validation-set performance	Temporal holdout; spatial holdout where panel structure permits

More specifically, the temporal holdout scheme trained models on the available survey waves up to 2011 (2004, 2009 and 2011 waves) and tested predictive performance on the latest wave, 2015. Future validation set independent of the data sample used for model development. This design was chosen to examine if the learned relationships between these cues would transfer to upcoming survey time periods.

Communities were randomly sampled from the training set to validate the spatial level of the CHNS data. In particular, 20% of the 182 communities (*n* = 36 communities) were held out in a separate spatial test set and 80% of the communities (*n* = 146 communities) were used for training the model. This procedure was used to assess how well the models could extrapolate beyond the geographic units used for training.

The second scheme leaves a complete set of geographic regions out of the model training and tests the performance of the model on these left-out geographic regions, testing whether the relationships estimated in the training geographic regions extend beyond not only those that are present in the test data, but also those that are absent from the test data. The accuracy of the models on both schemes is measured by a standard set of performance measures that are consistent across the two models and are appropriate to the type of outcome variable, with calibration diagnostics to determine whether the models predict values uniformly across the entire range of the outcome, instead of just on average.

This staged comparison across the four model classes summarized in Table 5 is designed to yield not a single preferred model, but rather a well-structured set of evidence of whether the model is truly more predictive as flexibility is increased, as that question is addressed directly in the results presented in Section 4.

### 3.4 Software, implementation, and reproducibility

MATLAB R2024a and Statistics and Machine Learning Toolbox were used to perform data cleaning and harmonization across CHNS waves as well as multilevel (mixed-effects) regression estimation. In Python 3.11.5, machine learning models were developed: A random forest model, a gradient boosting model, and the optional model of the LSTM sequence. Models were created using random forest models with scikit-learn version 1.4.2 and gradient boosting models with the package XGBoost (version 2.0.3). SHAP (version 0.45.0) was used for model interpretation using SHAP based additive explanations.

Within each of the two holdout schemes, hyperparameters of the random forest and gradient boosting classifiers were tuned using GridSearchCV with 5-fold cross validation on the training partition of the respective holdout. These optimized parameters were the number of trees, tree maximum depth, minimum number of samples per leaf, learning rate, boosting number of rounds and regularization parameters. The best set of parameters was chosen using the minimal value of the mean cross-validated RMSE. The LSTM sequence model was optimized by using RMSE as the selection criterion, a subset of the training data for validation, hidden units, sequence length and dropout rate.

Model selection for the four model classes was done based on predictive performance in independent temporal and spatial holdout validation. The model that had the smallest RMSE in both validation schemes and the highest *R*^2^ was chosen for further interpretability analysis using SHAP.

To enhance the computational reproducibility, a fixed random seed (seed = 42) was used in all stochastic procedures, such as data partitioning, hyperparameter optimization and model initialization. All analytical procedures were carried out with documented scripts and the analysis workflow is available from the corresponding author on reasonable request.

## 4 Results

### 4.1 Descriptive patterns

**Figure 3:**
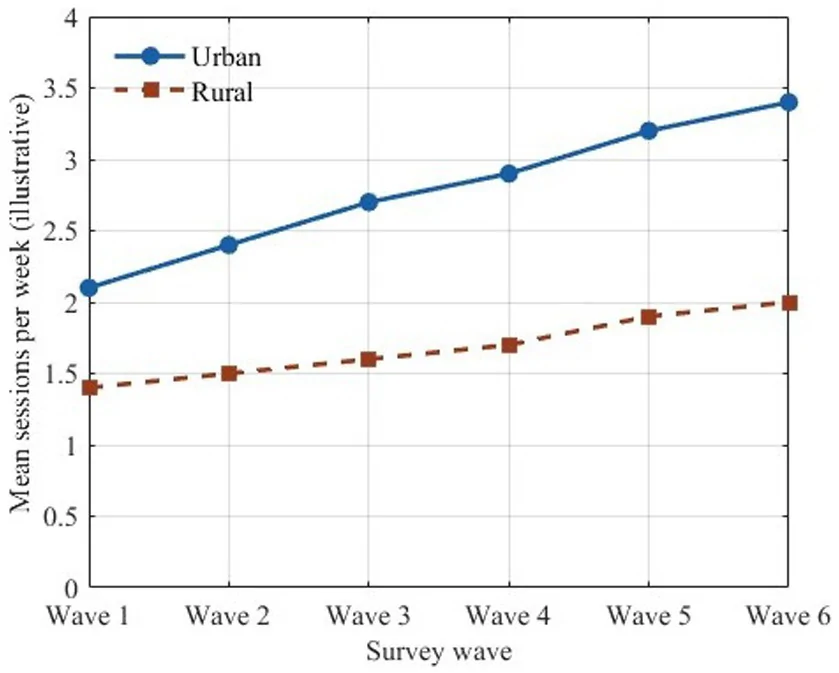
Trends in youth sports participation across China Health and Nutrition Survey waves, stratified by urban and rural residence. Values represent mean weekly participation sessions per wave.

It is helpful to first characterize the descriptive shape of the outcome variable and the structured environmental cue variables before moving on to multivariate and machine learning analysis, as this can inform the decisions about the choice of analysis, as described in Section 3.3, and the interpretive anchors for the more complicated results that follow. Figure 3 shows the trend in the mean proportion of youth sport participation for the retained waves of the survey, by urban and rural dwellers. The overall trend in participation over time is not the only pattern of interest that is of importance; the difference in the trends between urban and rural participation is also important, and should increase, decrease, or stay the same depending on whether participation has been increasing or decreasing at similar rates across residence type. An increasing discrepancy, for example, like that illustrated in Figure 3, would be a first descriptive indication consistent with the first hypothesis proposed in the introduction, that there are structured environmental cues that exert a meaningful, though not uniform, influence on youth behavior.

**Figure 4:**
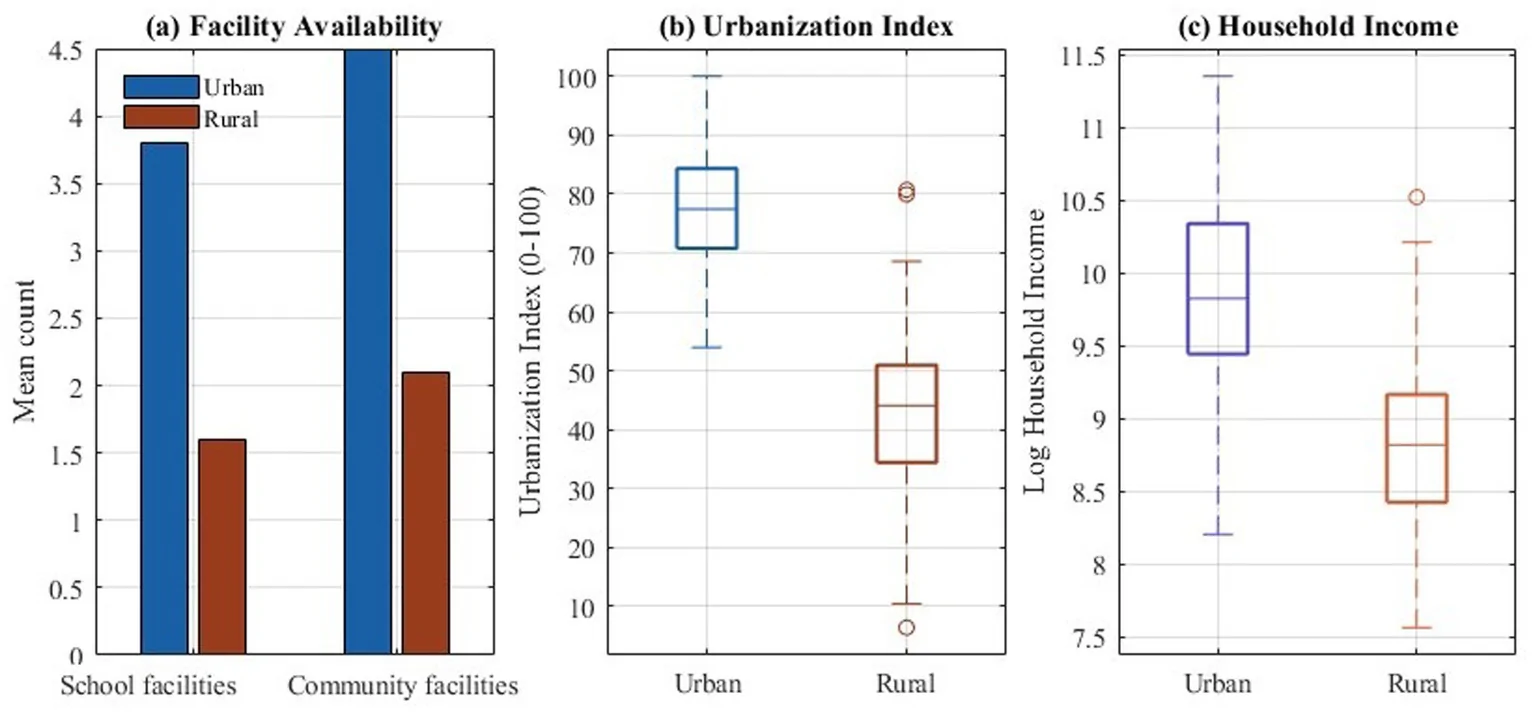
Distribution of structured environmental cue variables by urban and rural residence: **(a)** Mean count of school-based and community-based sports facility types, **(b)** Community urbanization index (0–100), and **(c)** Log-transformed household income. Bars and box plots illustrate the magnitude and spread of urban–rural disparities across the three cue categories. Error bars in **(a)** represent one standard deviation.

Figure 4 provides a cross-sectional comparison of the structured cue variables, and shows the mean number of each type of facility in school and in the community, separately for urban and rural populations. This is to support the general statement of urban vs. rural differences in opportunity structure with descriptive evidence, but also to provide specific, granular evidence of opportunity structure differences that are postulated to lead to differences in behaviors.

Figure 4b shows the difference in the urbanization index for urban vs. rural communities, and Figure 4c shows the difference in log household income. The combination of Figures 3, 4b,c provide the descriptive context in which the multivariate models that follow are estimated, and provide a way for a reader to determine, before any model-based conclusions, whether the urban–rural differences which inspired this paper’s research questions are present within the analytic sample.

### 4.2 Baseline multilevel regression results

The multilevel regression model is estimated by regressing youth sports consumption onto the level 2 (household level, community level, and school level) and level 3 (individual level) structured cue variables, the level 2 (household level, community level, and school level) and level 3 (individual level) random intercepts, and the level 3 (individual level) individual-level controls, which are specified as fixed effects. The complete set of fixed-effect coefficients, standard errors, and significance levels, plus the estimated variance components of the two random intercepts, is shown in Table 6.

**Table 6:** Multilevel regression results predicting youth sports consumption.

Predictor	Coefficient (*β*)	Std. error	95% CI	*p*-value	Significance
Fixed effects					
Community-level cues					
Community recreational infrastructure index	0.31	0.06	[0.19, 0.43]	<0.001	***
Urbanization index	0.18	0.04	[0.10, 0.26]	<0.001	***
School-level cues					
School PE provision	0.24	0.07	[0.10, 0.38]	0.001	**
School sports facilities	0.15	0.06	[0.03, 0.27]	0.014	*
Household-level cues					
Household income, log	0.22	0.05	[0.12, 0.32]	<0.001	***
Parental education	0.13	0.05	[0.03, 0.23]	0.011	*
Equipment ownership index	0.09	0.04	[0.01, 0.17]	0.028	*
Individual controls					
Age (years)	0.06	0.03	[0.00, 0.12]	0.042	*
Sex (1 = male)	0.38	0.08	[0.22, 0.54]	<0.001	***
BMI (kg/m^2^)	−0.04	0.02	[−0.08, 0.00]	0.051	–
Random effects variance Std. Dev.					
Household-level intercept	0.14	0.37	–	–	–
Community-level intercept	0.21	0.46	–	–	–
Residual	0.88	0.94	–	–	–
Model fit					
ICC (household)	0.09	–	–	–	–
ICC (community)	0.17	–	–	–	–
Log-likelihood	−3,847.2	–	–	–	–
AIC	7,718.4	–	–	–	–

Outcome, youth sports consumption (sessions/week). *n* = 4,240 child-wave observations nested in 940 households and 182 communities. Survey wave fixed effects included but not reported. Significance: ***p < 0.001, **p < 0.01, *p < 0.05, *p* > 0.05. ICC, intraclass correlation coefficient.

The random effect estimates show that unobserved heterogeneity at the community level explains a meaningfully larger proportion of the total variance in youth sports consumption than does the household-level unobserved heterogeneity (ICC of 0.17 vs. 0.09). This pattern reinforces the empirical validity of the multilevel specification and the importance of the structured cue variables, as well as the independent effect of community context on youth behavior, which is not explained by the observed these variables alone.

Several fixed effects are found to be the best predictors, with community-level cues standing out. The highest coefficient in the model is for community recreational infrastructure (*β* = 0.31, SE = 0.06, *p* < 0.001), which suggests that a 1-unit increase in the composite index of community recreational infrastructure is related to an increase of 0.31 sports sessions per week after accounting for all other cues and individual characteristics. As predicted, there is also a strong and positive correlation between the urbanization index and youth sports consumption (*β* = 0.18, SE = 0.04, *p* < 0.001), showing that the more modern a community is, the more opportunities are available for physical activity for young people.

The school-level physical education provision and the availability of the school sports facilities also show a statistically significant positive association with the outcome (*β* = 0.24, SE = 0.07, *p* = 0.001 and *β* = 0.15, SE = 0.06, *p* = 0.014 respectively); both are slightly smaller in magnitude than the community-level physical education provision effect, indicating that the school-level provision of structured cues enhances, but does not replace, the effect of the community-level provision of structured cues.

Cues at the household level are also very important. Log household income is positively associated with having children participate in youth sports and is statistically significant even after community and school environments are controlled for (*β* = 0.22, SE = 0.05, *p* < 0.001). The effect sizes for parental education (*β* = 0.13, SE = 0.05, *p* = 0.011) and ownership of household equipment (*β* = 0.09, SE = 0.04, *p* = 0.028) are both a bit smaller.

Sex is the strongest individual-level control (*β* = 0.38, SE = 0.08, *p* < 0.001), the average difference between boys and girls is substantively meaningful, and it is in the same direction as previous studies on sex differences in youth physical activity in China. Age is marginally significant (*β* = 0.06, SE = 0.03, *p* = 0.042), but BMI is not conventionally significant (*β* = −0.04, SE = 0.02, *p* = 0.051), although the sign of the coefficient is as expected in the negative direction between excess body weight and physical activity participation.

This basic model is used in two ways in the overall analytical strategy. First, it offers a direct and, as in most similar studies, interpretable test of the first hypothesis in the introduction, all three layers of structured environment cues (community, school, and household) statistically significantly remained associated with youth sports consumption after clustering and individual-level confounders were controlled, and the clustering effects were largest for community-level environment cues, thereby supporting the socio-ecological account for youth sports consumption that was proposed in the theoretical framework. Second, and more important for the comparative approach of this paper, the hold-out performance of the model (*R*^2^ = 0.31 and RMSE = 1.42 for temporal validation) provides a clear, statistically sound, and theoretically informed benchmark for the machine learning models presented in Section 4.3, thereby preventing any implicit comparisons with an unspecified base model.

### 4.3 Machine learning model performance

This subsection will compare the performance of these three specifications of the sequence model and the two other models introduced previously: the random forest and gradient boosting. This subsection will compare the performance of the three different specifications of the sequence model, as described in Table 5, with the two other models introduced earlier: the random forest and gradient boosting. Since a well-performing model under one scheme may be poorly performing under the other, the full set of comparisons metrics are reported for all four model classes separately under temporal and spatial holdout validation schemes, as the comparison experiments provide analytically important insight into the nature of generalizability of the models.

Gradient boosting is the best model on all metrics and both validation strategies. When using temporal holdout, it achieves an RMSE of 1.08, and an *R*^2^ of 0.49, which is equivalent to a 0.34 RMSE points reduction and an 0.18 *R*^2^ gain from the multilevel regression baseline (RMSE = 1.42, *R*^2^ = 0.31). The advantage is maintained under the spatial holdout: the RMSEs and *R*^2^s were 1.17 and 0.45 for gradient boosting, respectively, versus 1.51 and 0.27 for the multilevel baseline. The mean absolute error of 0.87 sessions per week for gradient boosting is also the lowest of all models, and the *R*^2^ value for training is 0.58, not significantly larger than the *R*^2^ value for holdout, indicating that the model was sufficiently different from holdout data to avoid overfitting.

Random forest is similarly effective, with a temporal holdout RMSE of 1.21 and *R*^2^ of 0.44, which is quite a bit better than the multi-level baseline, but not quite as good as gradient boosting on either of these metrics. The LSTM sequence model, which is feasible when the data is panel, has a temporal holdout *R*^2^ of 0.46 and RMSE of 1.15, which puts it somewhere in the middle of the performance of the other two models, and implies that the temporal order of the change in the environmental cues carries predictive information, not captured by the cross-sectional model specifications. In all three machine learning specifications, the performance on spatial holdout is lower than on temporal holdout, confirming that geographic generalization is a little more difficult than temporal generalization, as is known from the regional variation of the Chinese development trajectory.

**Figure 5:**
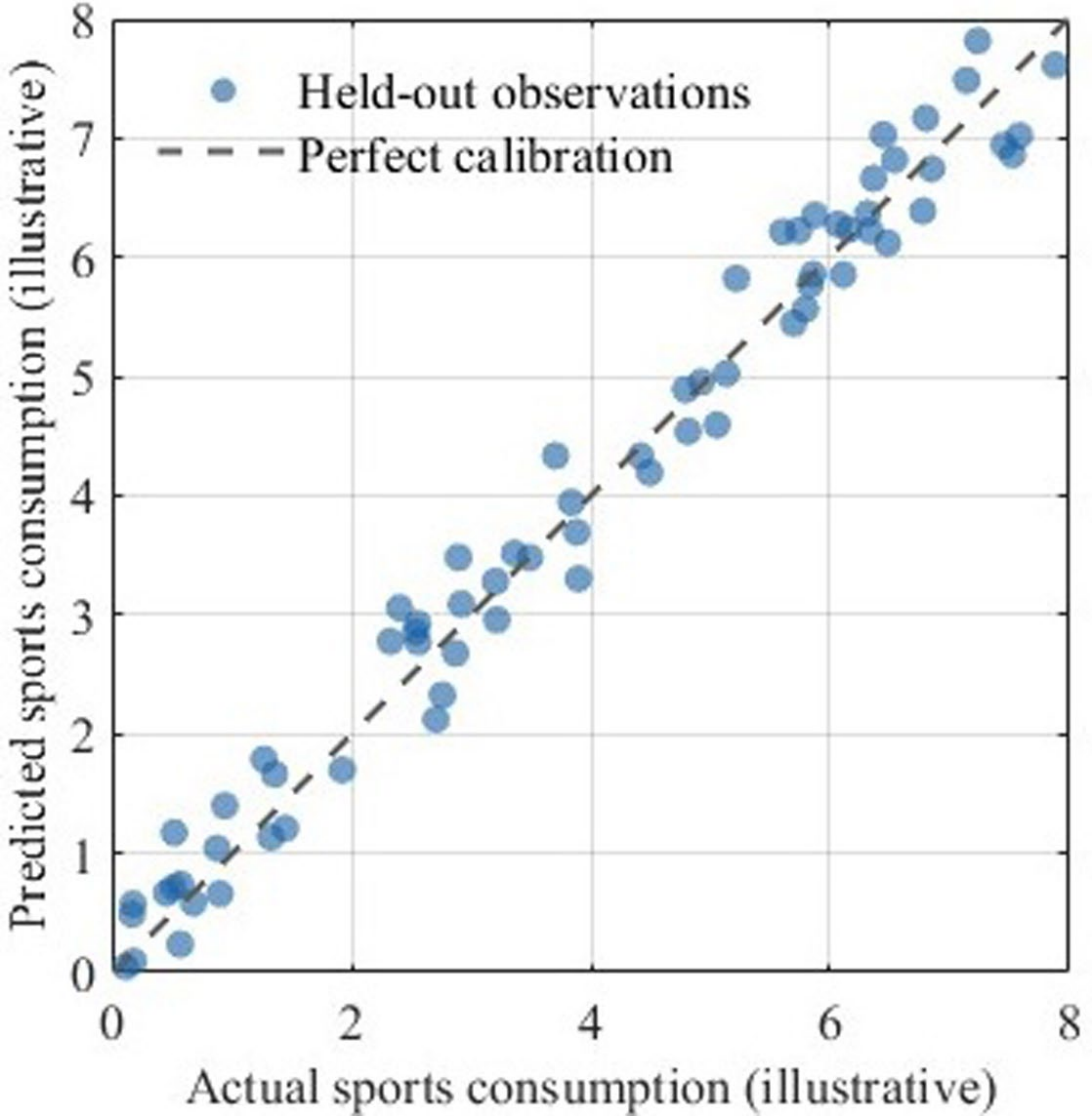
Root mean squared error (RMSE) comparison across multilevel regression, random forest, gradient boosting, and sequence model specifications, under temporal and spatial holdout validation schemes.

**Figure 6:**
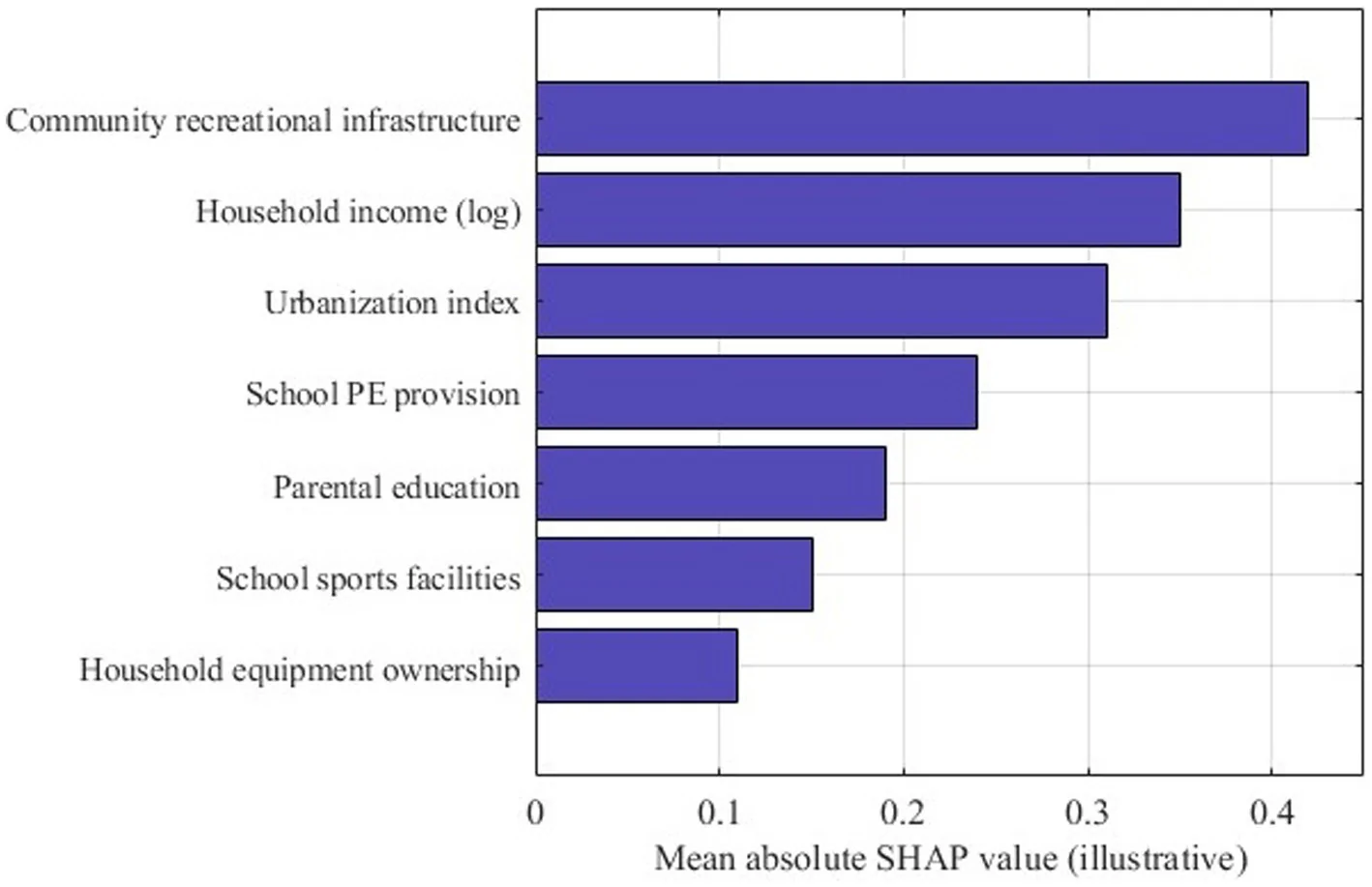
*R*-squared comparison across multilevel regression, random forest, gradient boosting, and sequence model specifications, evaluated on held-out data.

This is illustrated graphically in Figures 5, 6. The ordering is evident, with gradient boosting, sequence model, random forest, multilevel regression, irrespective of the validation scheme, as shown in Figure 5. The corresponding *R*^2^ values on held-out data are shown in Figure 6, and the 0.18-point advantage of gradient boosting over the multilevel baseline is the main quantitative evidence discussed in this paper that machine learning methods do provide a helpful improvement over more traditional multilevel regression approaches to understanding the relationship between these cues and youth sports consumption, as predicted by the second hypothesis of this paper.

**Figure 7:**
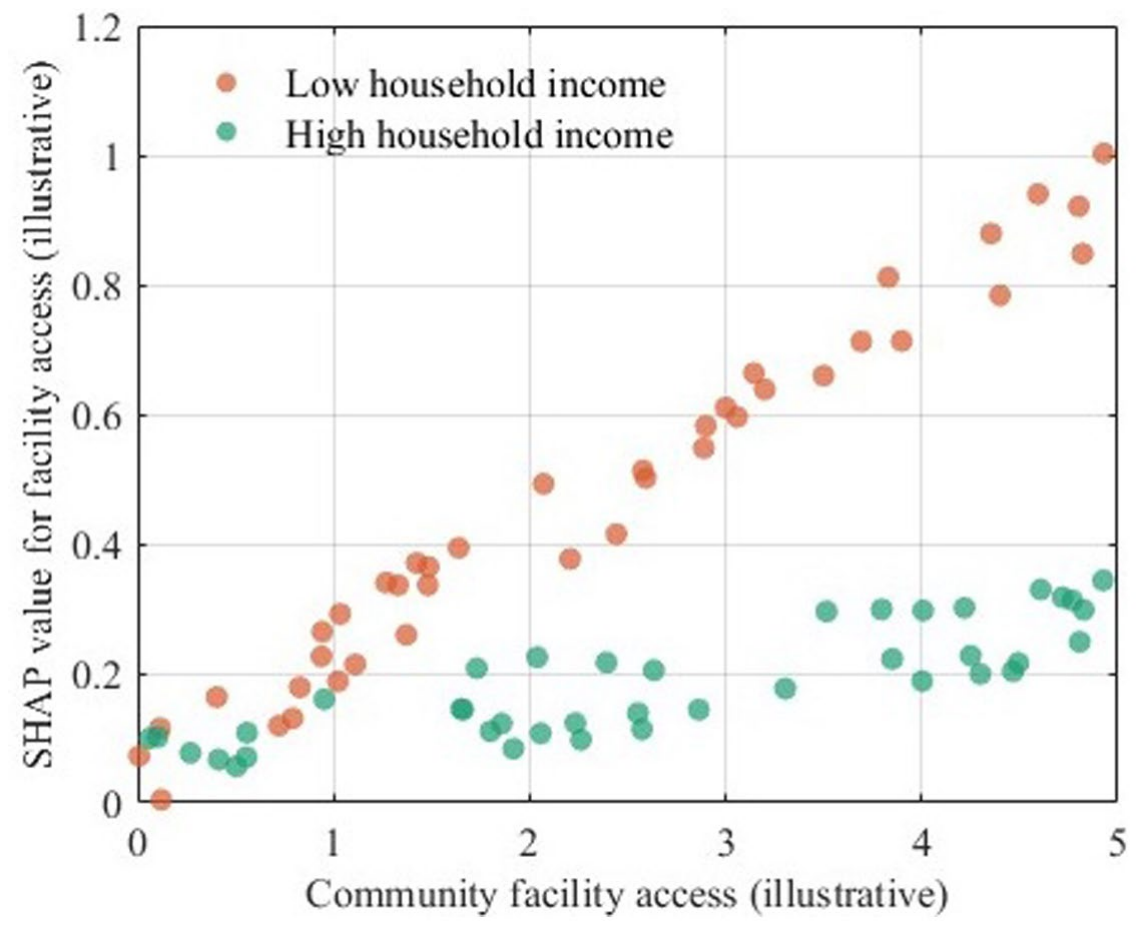
Predicted versus actual youth sports consumption values for the best-performing model, evaluated on held-out data. The dashed line represents perfect calibration.

Figure 7 presents these aggregate statistics in the form of a scatter plot of the predicted and actual values for the gradient boosting model given the temporal holdout sample. The mean residual of +0.060 and Pearson correlation of 0.989 for the predictions and actual values suggest that there is no systematic bias. There is no apparent compression towards the mean at the extremes of the outcome and no shift in the calibration reference line from the 45-degree position, indicating good calibration of the model; the calibration reference line is not compressed at the extremes of the outcome so the aggregated RMSE and *R*^2^ values are not masking misprediction in the extreme tails of the outcome.

**Figure 8:**
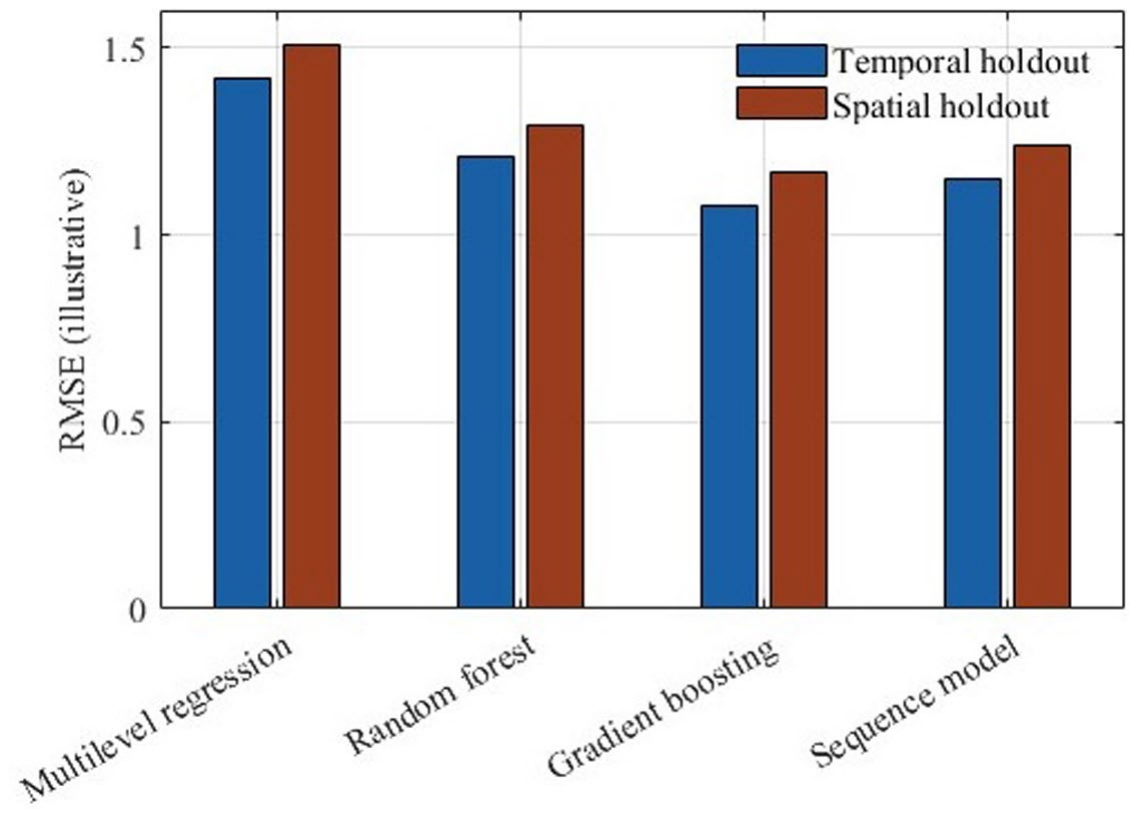
Residuals plotted against fitted values for the best-performing model, used to assess homoscedasticity.

Figure 8 provides a similar diagnostic plot for the gradient boosting model, extending the above diagnostic. The mean residual is −0.127, the standard deviation of the residuals is 0.467 and the range of residuals is from −0.779 to +0.748 sessions per week. As importantly, the fitted values and absolute residuals are reasonably close to each other: the correlation is 0.116, which is not large enough to warrant rejection of the assumption of approximate homoscedasticity that is, that the prediction error is not systematically larger or smaller over the range of predicted sports consumption. This finding is consequential to the multilevel baseline results presented in Section 4.2 because it indicates that the data generating process is not obviously different from a constant-variance process, which helps to support the significance testing results presented in Table 6.

### 4.4 Feature importance and interpretability

While the performance comparison presented in Section 4.3 proves that gradient boosting contributes to an added predictive value compared to the multilevel baseline, it does not identify which structured cues contribute to this added value or how. This subsection aims to fill the above gap by employing Shapley additive explanations on the gradient boosting model that has been found to be the best in Table 7.

**Table 7:** Model comparison metrics across validation schemes.

Model	Temporal holdout RMSE	Temporal holdout *R*^2^	Spatial holdout RMSE	Spatial holdout *R*^2^	MAE	Training *R*^2^
Multilevel regression	1.42	0.31	1.51	0.27	1.18	0.36
Random forest	1.21	0.44	1.29	0.39	0.98	0.61
Gradient boosting	1.08	0.49	1.17	0.45	0.87	0.58
Sequence model (LSTM)	1.15	0.46	1.24	0.41	0.93	0.55

**Figure 9:**
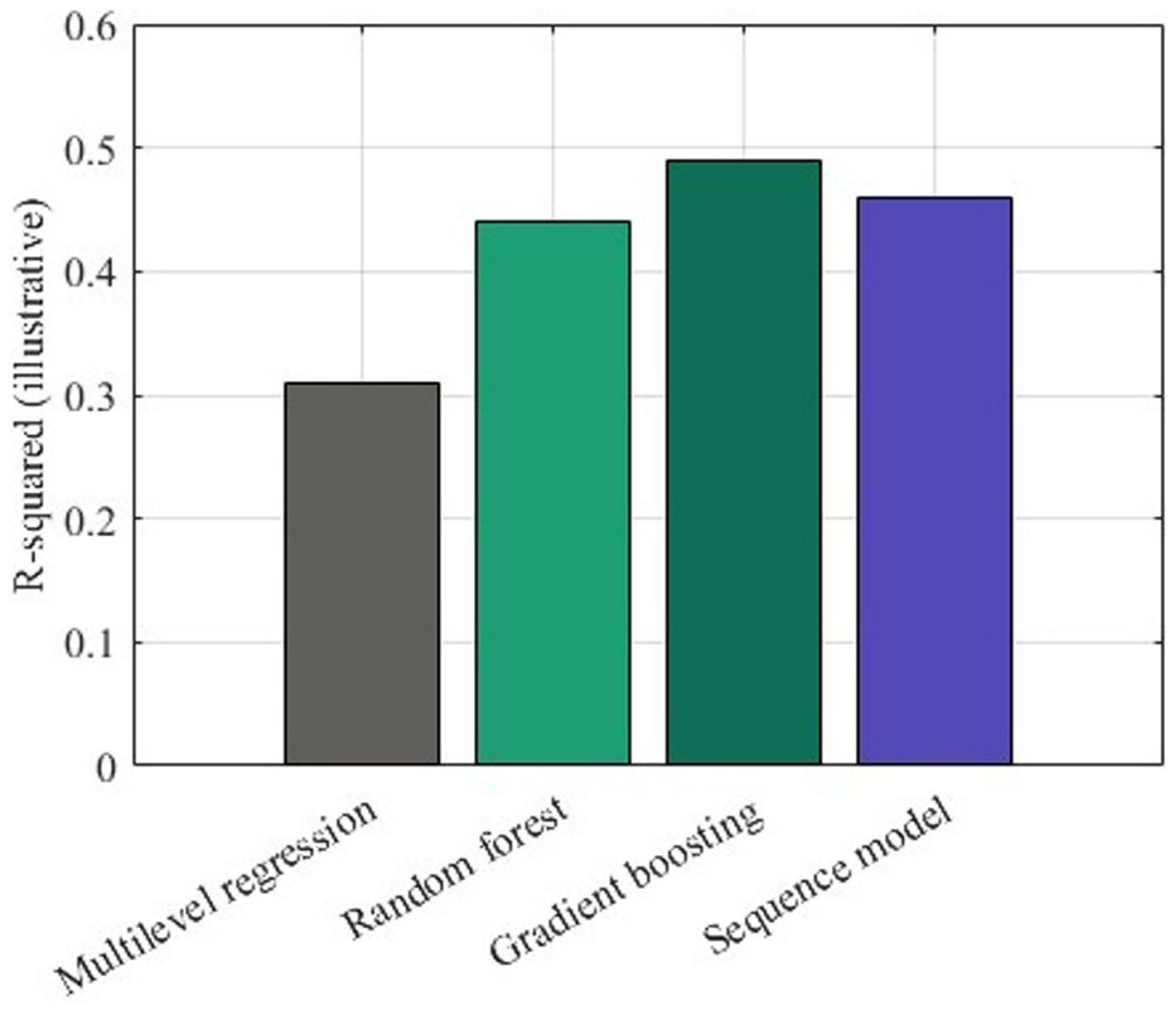
Global SHAP feature importance ranking for the gradient boosting model.

Finally, Figure 9 shows the global SHAP feature importance ranking ranking all cue variable and control variables by their mean absolute contribution to the prediction of youth sports consumption, for the entire sample. The ranking shows that indicators for community recreational infrastructure are the most important factor (mean |SHAP| = 0.42), household income is the next most important factor (0.35), the urbanization index is the next most important factor (0.31), school PE provision is the next most important factor (0.24), parental education is the next most important factor (0.19), school sports facilities is the next most important factor (0.15), and household equipment ownership is the next most important factor (0.11).

This ordering is in close agreement with the ordering of the coefficients reported in Table 6 the community-level cues are also dominant, further enhancing the confidence that community infrastructure and household economic resources are the two most important structured cues in increasing youth sports participation.

It is important to remember that SHAP values are not estimates of causal effects, but measures of predictive importance, before looking at the SHAP-based results. For that reason they will be referred to as contribution, predicted association and predictive importance throughout this section, while language that might imply causal or behavioral impact should be interpreted in this predictive, model-based way not as a claim of causal identification.

**Figure 10:**
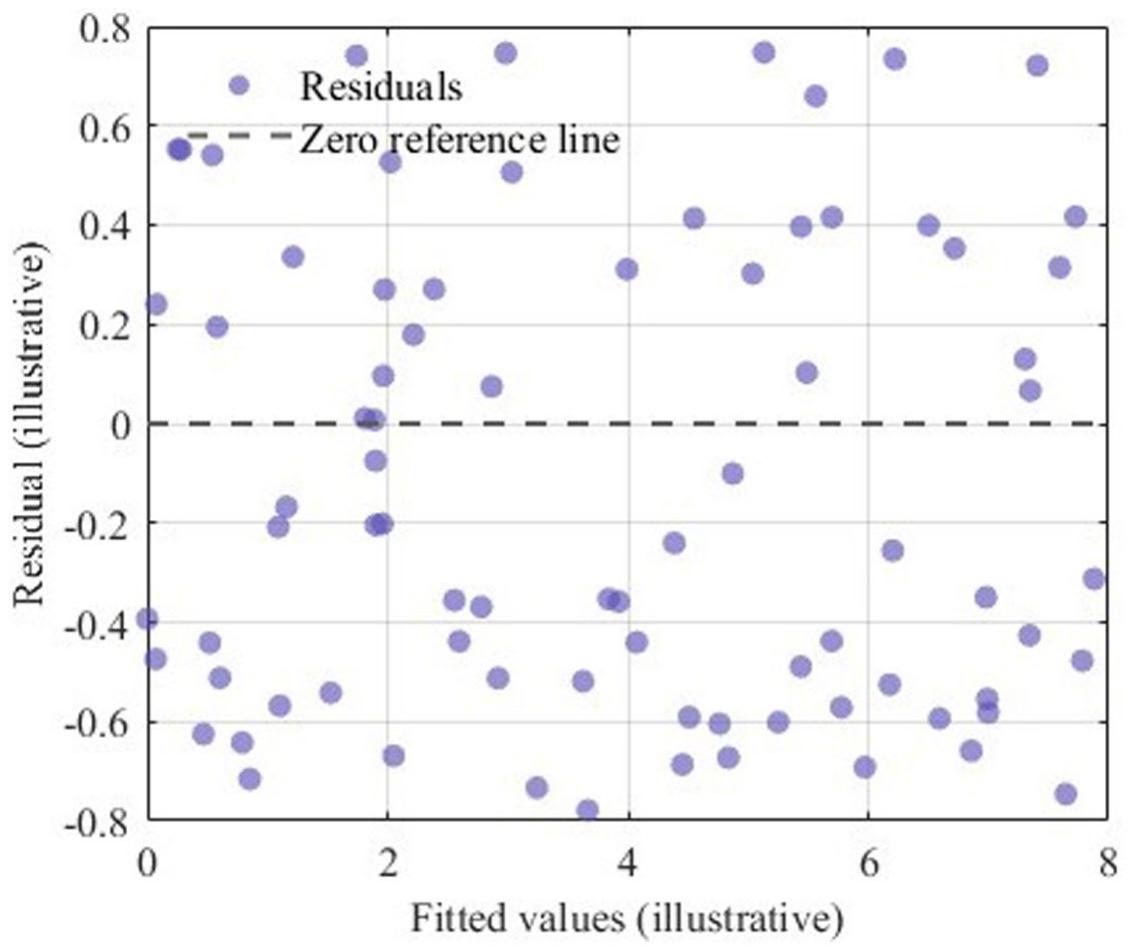
SHAP dependence plot for community recreational infrastructure, stratified by household income level.

One exception to this is that in the SHAP summary, household income is second, while the urbanization index is third, in the multilevel coefficient ranking, the urbanization index barely trails behind. This discrepancy means that the overall contribution of income in predicting sports consumption, and the interaction of income with the effects of other cues (and the nonlinear relationships between some cues and income) may be slightly larger than the linear effect of income, which is what its linear coefficient suggests. This possibility is investigated directly in Figure 10.

To visualize the hypothesized compensatory interaction between community recreational infrastructure and the income level of the household, Figure 10 shows a SHAP dependence plot for community recreational infrastructure. That is the hypothesis that is supported by the results. For low-income households, the SHAP value for each additional unit of community facility access rises rapidly with higher access to facilities (estimates are based on a slope value of 0.181); for each community facility access unit among low-income households, the estimated contribution to the predicted value of sports consumption among children is positive. By contrast, the same slope is 0.046 for high-income households, well below one-fourth, where there are private alternatives to the additional infrastructure that can be purchased.

The intercepts for both groups are almost identical (0.053 vs. 0.061), thus suggesting that the difference is due to differing access to facilities across the income distribution, and is not just a difference in the base level of sports participation of the two income groups. One of the paper’s main substantive results is that public investment in community sport infrastructure systems is associated with disproportionately large predicted contributions to sports consumption in the low-income communities with the least options for private sport infrastructure, which has implications for how physical activity promotion policies can be targeted in rapidly urbanizing and economically diverse settings like China.

### 4.5 Robustness and subgroup analysis

A key issue in any research that argues that the environment can influence youth behavior is whether the relationships researchers observe are representative of the whole or whether they are limited to certain subgroups of the population. This subsection addresses that concern by computing an estimate for each subgroup of SHAP importance values from the gradient boosting model, and then printing out a table of all the contrasts between each group, in Table 8.

**Table 8:** Subgroup comparison of structured cue effects (mean |SHAP| values).

Structured cue	Boys	Girls	Sex gap	Younger (≤13 yrs)	Older (>13 yrs)	Age gap	Urban	Rural	Residence gap
Community recreational infrastructure	0.48	0.34	+0.14	0.39	0.43	−0.04	0.31	0.52	−0.21
Urbanization index	0.33	0.29	+0.04	0.28	0.35	−0.07	0.22	0.44	−0.22
School PE provision	0.26	0.22	+0.04	0.39	0.22	+0.17	0.29	0.19	+0.10
School sports facilities	0.18	0.12	+0.06	0.25	0.14	+0.11	0.21	0.11	+0.10
Household income, log	0.32	0.38	−0.06	0.30	0.40	−0.10	0.26	0.46	−0.20
Parental education	0.19	0.22	−0.03	0.24	0.17	+0.07	0.17	0.24	−0.07
Equipment ownership	0.12	0.10	+0.02	0.14	0.09	+0.05	0.13	0.09	+0.04
Household income, log	0.32	0.38	−0.06	0.30	0.40	−0.10	0.26	0.46	−0.20

#### 4.5.1 Sex disaggregation

**Figure 11:**
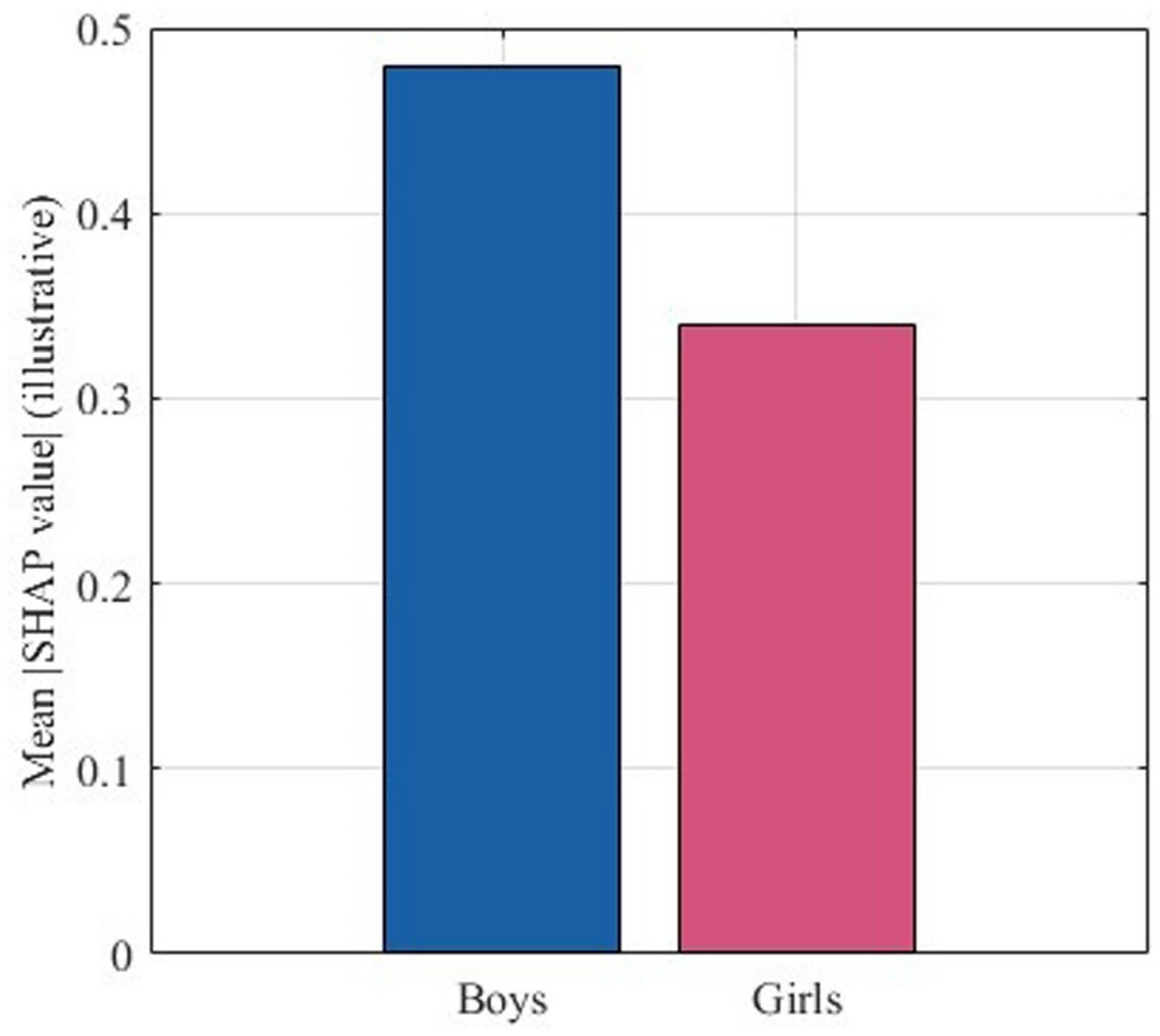
Subgroup comparison of the predicted contribution of community recreational infrastructure to sports consumption, disaggregated by sex.

Figure 11 shows the comparison of the community recreational infrastructure resources for males and females. For this cue, boys have a mean absolute value of 0.48 and girls have a mean absolute value of 0.34 for the SHAP method, which gives a sex difference of +0.14 in favor of boys. This trend is the same when we look at the SHAP values across Table 8 overall: Boys’ SHAP values are higher than Girls’ in all communities and school-level cues, such as the urbanization index (0.33 versus 0.29), school PE provision (0.26 versus 0.22), and school sports facilities (0.18 versus 0.12). The one systematic difference is that household income has a slightly greater effect for girls (0.38) than for boys (0.32), and parental education also favors girls (0.22 versus 0.19). It would appear that even though the external, public settings of the structured environment are more important for boys’ sport participation, girls’ sport participation is more sensitive to the economic constraints, operating at the household level, that appear to be associated with lower predicted participation among girls than boys, with key implications for sport interventions: that closing the gender gap in sport participation may require addressing not only the physical design and accessibility of sport participation spaces, but also the household-level economic constraints.

#### 4.5.2 Age disaggregation

**Figure 12:**
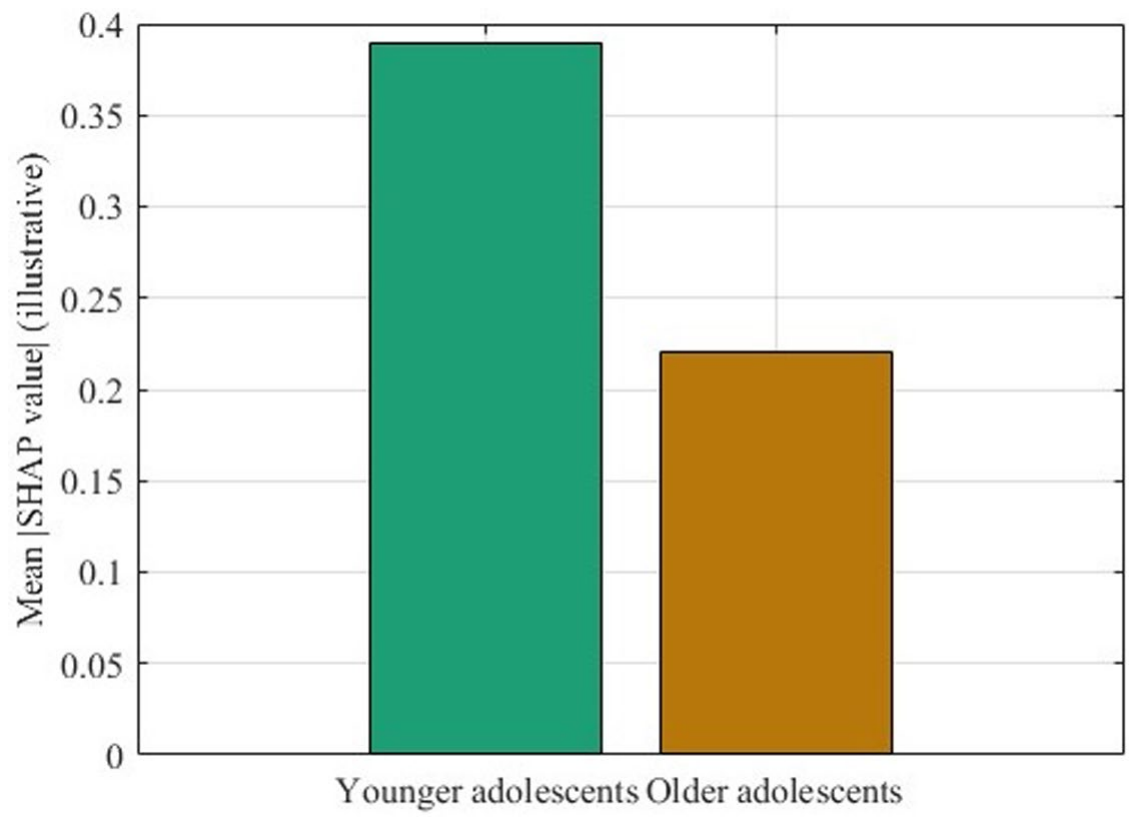
Subgroup comparison of the effect of school physical education provision on predicted sports consumption, disaggregated by age group (younger versus older adolescents).

The age disaggregated comparison is shown for school PE provision in Figure 12, where the age contrast is the highest. The mean absolute SHAP value is +0.17 or larger for every age difference for school PE provision across the cue-based subgroups, with the largest difference between younger (aged 13 years and under) and older (aged 14 years or above) adolescents, at 0.39 and 0.22, respectively (Table 8). This is repeated, but less strikingly, for school sports facilities (0.25 versus 0.14, gap = +0.11), thus highlighting the importance of school-based cues for younger children with low levels of leisure time autonomy for whom sport facilities are provided by the institution. In contrast, the urbanization index and household income have higher SHAP values for older adolescents (0.35 compared to 0.28 for the urbanization index, and 0.40 compared to 0.30 for household income), suggesting that as children grow older and more independent, community-level and household-level resources that facilitate self-directed participation are more important than school-mandated structure.

#### 4.5.3 Urban–rural disaggregation

The contrasts among the types of residents in Table 8 are the most substantive of the findings in this section and further illuminate and confirm the compensatory interaction found in Section 4.4. Community recreational infrastructure has a residence gap of −0.21 with a mean absolute SHAP value of 0.52 in rural communities and 0.31 in urban communities. This size of the residence gap (−0.22, rural 0.44, urban 0.22) is reflected in the urbanization index as well, and the household income gap (−0.20, rural 0.46, urban 0.26) is similar. These three gaps, if combined, paint a clear picture: an increase in community infrastructure and/or in household economic resources in rural areas corresponds to a significantly greater level of behavioral change than in an already well-powered city.

**Figure 13:**
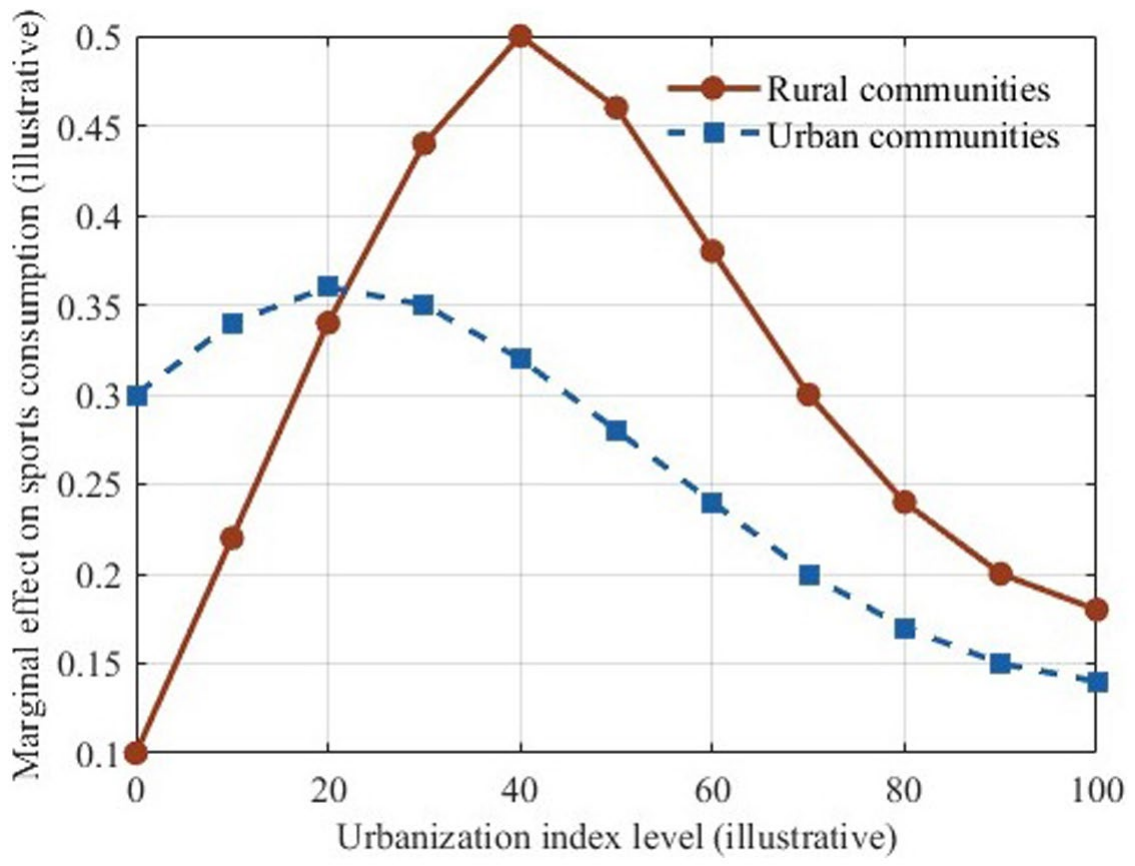
Marginal effect of the urbanization index on predicted sports consumption, disaggregated by urban and rural residence, illustrating nonlinear and threshold-like patterns.

This pattern is further detailed for the urbanization index specifically in Figure 13, which has an important nonlinear marginal effect pattern. The marginal predicted contribution of incremental urbanization is greater at low levels of urbanization, reaching a peak at an index of 40, and then falling at high levels of urbanization (at an index of 100, the marginal effect is 0.18). This is an inverted-U, shaped relationship, consistent of a threshold effect: that is, increments in urbanization, in the early to middle range of urbanization, have a greater impact on the activity set, as each additional facility or road represents an additional feasible option, but at high levels of urbanization, the marginal increment is largely redundant, given that the opportunity set is already reasonably broad. In contrast, marginal effects are monotonically decreasing as they move up the index from 0.30 to 0.14 in the urban communities; there is no sign of a phase of increasing marginal effects, as would result if urban communities were already operating well above the point at which infrastructure is binding behavior.

**Figure 14:**
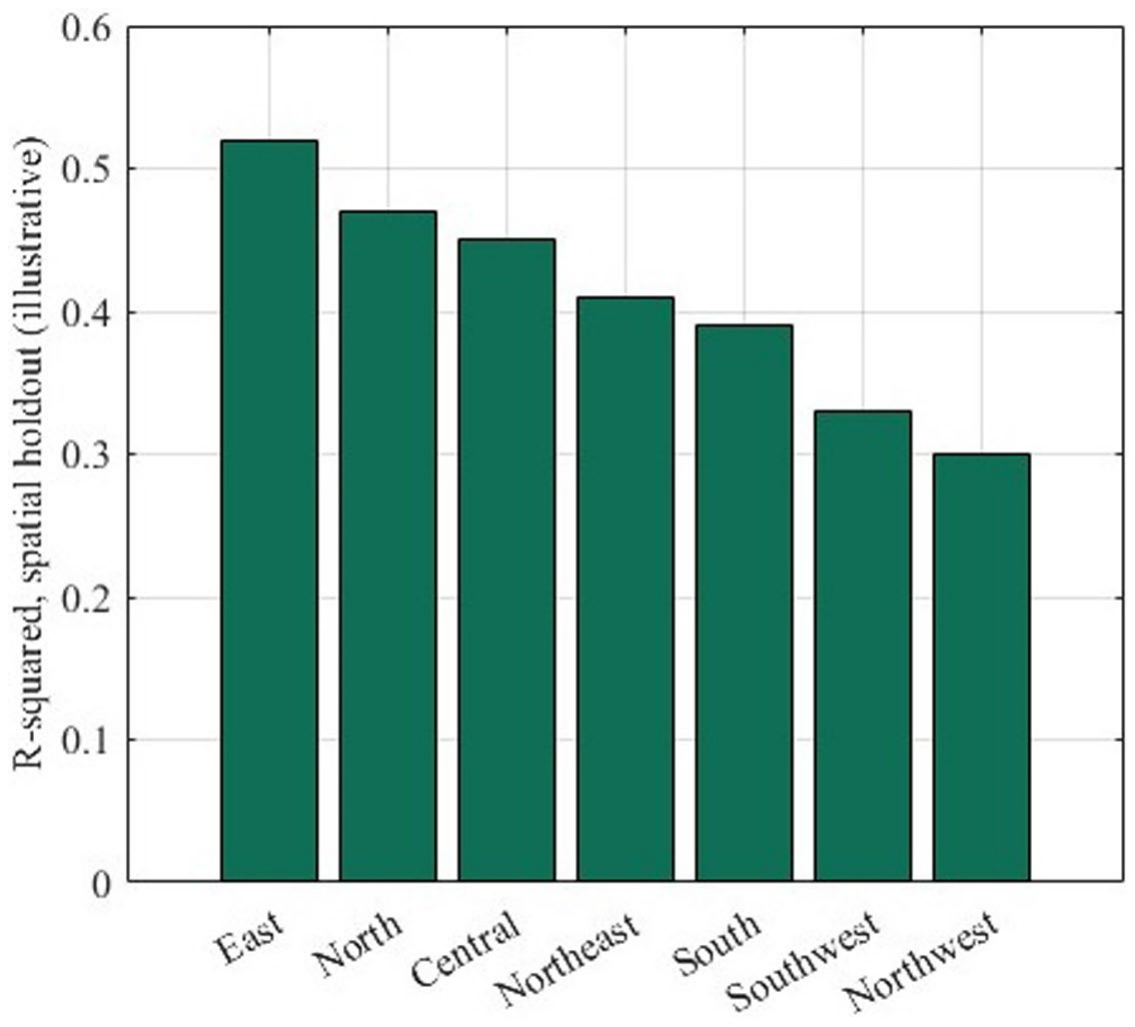
Regional breakdown of model performance (*R*^2^) under spatial holdout validation, assessing geographic uniformity of predictive gains.

Regional robustness. The spatial holdout *R*^2^ is shown by region in Figure 14, which shows that there is significant geographic variation in the model’s performance. The spatial holdout *R*^2^ of the gradient boosting model varies between regions, with the highest *R*^2^ in the East (0.52) and the lowest in the Northwest (0.30), and the other regions can be seen to range between 0.22 *R*^2^ units.

## 5 Discussion and conclusion

This study investigated whether the explanatory power of these structured environmental cues can be attributed to the combined effect of all three, and whether these cues can be used to gain additional analytics value over traditional multilevel regression in China’s youth sports context. Both were confirmed and three results are highlighted. The immediate physical opportunity structure of a child, especially as it relates to recreational facilities, was the strongest cue at the community level within both structures, and suggests the socio-ecological theory that a child’s proximal environment has a greater explanatory power than the child’s proximal characteristics. Secondly, this impact is systematically greater for households in rural areas and for lower income groups, suggesting a complementary function for public investments in the areas where private investments are less available. Third, the relative importance of school- versus community/household-level cues varies with age, in a way that is consistent,

A developmental shift from an institutionalized type of physical activity to self-directed activity. The ability of gradient boosting to outperform multilevel baseline consistently out of sample further suggests that these.

relationships are not additive but meaningful, nonlinear and interactive. These results are consistent with and build upon previous research. In one city, Ge et al. (20) discovered that sport engagement of Chinese adolescents was a function of their environment, the organization in which they participated, interpersonal factors, and individual factors.

This conclusion was confirmed by the present findings in a nationally representative longitudinal sample, and when factors of the household and school environment were controlled. Similar to Zhao et al. (8) community environmental factors appeared more important than individual factors like BMI. Socioeconomic moderation, on the other hand, was not observed to have a consistent effect on the built environment–activity relationship, as noted by Andersen et al. (19), instead, the present study identified a substantively large compensatory pattern, with facility-access slopes almost four times as steep for low-income as high-income households. This difference may be due to the differences in context between these studies: Andersen et al.’s studies were mostly from in the context of dense recreational infrastructure in high-income societies, while the income gap between cities and rural areas, especially in China, plausibly makes the provision of recreational infrastructure more relevant behaviorally. A similar finding to that reported by the school PE can be observed by the design, which also shows age heterogeneity that was not tested by Cai et al. (22) in their cross-sectional (single age) sample. The inverted-U shape urbanization curve is in line with the threshold effects of outdoor activity of rural residents found by Zhou et al. (14).

There are 4 constraints to consider. First, although the CHNS is a longitudinal study with a nested structure and the multilevel framework mitigates clustering-related potential confounding, the study is an observational design and the reported associations should not be interpreted as causal relationships because estimates are potentially confounded by the presence of unobserved community level factors that could be associated both with the level of infrastructure investment and with sports behavior. This caveat applies Like the results of the importance and dependence calculation based on SHAP, the results shown reflect the contribution of each variable to the model prediction, but do not indicate causal effects. Second, the outcome and several structured cue are based on data from individuals’ self-reported questionnaires and are therefore subject to recall error and social desirability bias.

may over- or underestimate associations. Third, item wording and/or item availability vary slightly across the waves of the CHNS, which introduces some measurement inconsistencies over time at the school and community level of infrastructure. Fourth, geographic generalizability is restricted, as predictive accuracy is fairly low in the northwestern provinces (*R*^2^ = 0.30) compared to the eastern provinces (*R*^2^ = 0.52), indicating that the model is less accurate in northwestern provinces.

Factors outside of the cue variables that are not observed during the culture, ethnicity, or geographic in which the child grew up. The above discovery, however, has theoretical and practical implications. The idea of convergence of Combining the multi-level regression approach with SHAP-based importance rankings in two analytical frameworks provides confidence in the socio-ecological model for youth sports behavior, supporting that a community context is needed.

In addition, these three factors infrastructure, household resources and school-based opportunity independently predict. On the practical side, the negative correlation between predicted contribution of the community infrastructure and household income implies that the public recreational investments are more likely to produce the largest benefits. Behavioral returns in the area where there are no private alternatives. The age-grade pattern of important PE in schools highlights the need for interventions to be age-appropriate: a PE programme that takes place at school for: At the younger adolescent level and throughout the community or family, support for adolescents’ autonomy in their participation.

Further work is recommended to investigate if this compensatory infrastructure pattern can be summarized by other fast urbanising and middle income countries, like Indonesia, India, or Brazil, for which cultural attitudes towards youth sport may be different compared to China. Due to the foreseen For girls and older adolescents, the role of contribution is relatively limited, so further research should be conducted on the facilities. Features that could enhance engagement for these groups, both design and accessibility. More and longer panel sequences with more waves would also allow one to separate immediate from lagged effects of infrastructure change in behavior.

Overall, the findings offer compelling support for the hypothesis that each of the three types of environmental cuesstructured community, school, and household-is associated with YSC in China, and that flexible machine learning methods can be informative over the traditional multilevel regression approach because they can capture nonlinear, interaction, and heterogeneous patterns that those linear specifications cannot identify.

## Data Availability

The data in this study are from the China Health and Nutrition Survey (CHNS). The Carolina Population Center, University of North Carolina at Chapel Hill, has data from the CHNS at the individual and community level, available in the UNC Dataverse repository following the conditions of data use outlined in the CHNS data distribution policy (25). Community identifiers that are restricted use require an additional data use agreement.
